# Female health-care providers’ advocacy of self-sampling after participating in a workplace program for cervical cancer screening in Ghana: a mixed-methods study

**DOI:** 10.1080/16549716.2020.1838240

**Published:** 2020-11-17

**Authors:** Anna-Lisa Behnke, Amrei Krings, Comfort Mawusi Wormenor, Priscilla Dunyo, Andreas M. Kaufmann, Joseph Emmanuel Amuah

**Affiliations:** a Clinic for Gynaecology (CBF), Laboratory for Gynaecologic Tumor Immunology, Berlin Institute of Health, Charité-Universitätsmedizin Berlin, Freie Universität Berlin, Humboldt-Universität zu Berlin, Berlin, Germany; b Department of Obstetrics and Gynecology, Catholic Hospital Battor, Battor, Ghana; c University of Ottawa, Ottawa, Canada

**Keywords:** LMIC, human papillomavirus, qualitative research, acceptability, cervical cancer screening advocacy

## Abstract

**Background:**

Cervical cancer is the second most common cancer among Ghanaian women and screening coverage is low. ACCESSING is a cross-sectional study investigating human papillomavirus (HPV) prevalence via self-sampling in rural communities of the North Tongu district in Ghana. Female health-care providers (HCPs) were invited to self-collect a cervicovaginal sample with a commercial sampler in order to acquaint themselves with the sampling method.

**Objective:**

This study set out to explore female HCPs’ perceptions, advocacy for, and implications of self-sampling with the aim of enhancing self-sampling acceptability in the targeted screening population.

**Methods:**

A mixed-methods approach was used, consisting of (a) a survey among 52 female HCPs working in a district hospital and (b) 10 one-to-one semi-structured interviews with purposefully sampled HCPs.

**Results:**

The quantitative analysis of the survey (n = 52) showed that, among HCPs who took the sample themselves (50/52), all found it ‘Easy’ or ‘Very Easy’ and felt ‘Very Comfortable’ or ‘Comfortable’. 82.7% indicated that they would undertake screening more often, and 98.1% indicated they would prefer self-sampling, if cervical cancer risk is as reliably determined as by clinician-directed cytobrush sampling. All interview participants (n = 10) indicated that they appreciated the program and would recommend the screening to their patients and/or family members and neighbours. Common reasons for preferring self-sampling were less (anticipated) pain compared to speculum examination and more privacy.

**Conclusions:**

Self-sampling for cervical cancer screening is highly acceptable to female HCPs. Setting up a workplace screening program that entails the option of self-sampling could create greater awareness and positive attitudes among HCPs to educating their patients, families, and neighbours on cervical cancer risks and motivate HCPs to advocate for women’s participation in screening.

## Background

In 2018, there were an estimated 570 000 cervical cancer cases and 311 000 cervical cancer deaths worldwide, approximately 90% of which occurred in low- and middle-income countries [1]. Cervical cancer is the second most common cancer and the leading cause of cancer-related deaths in women in Ghana [2]. The country’s crude incidence rate of 21.3 per 100,000 women annually and crude mortality rate of 14.3 per 100,000 women are high in comparison to estimates for high-income countries**As defined by the World Bank. (11.6 and 4.6 per 100,000 women) and low-income countries* (18.4 and 13.4 per 100,000 women) and higher than estimates for Western Africa (16.8 and 12.4 per 100,000 women) [2]. The majority of women present with late-stage cervical cancer [3]. Ghana currently does not have an organized national screening program [4,5] and the rate of cervical cancer screening by cytology is estimated at 2.8% (range 0.8–8.5%) [2,4–6]. There are limited data on uptake of screening, especially in rural regions. Currently, visual inspection with acetic acid (VIA) and cytology-based screening are only available in a few specialized hospitals [7,8].

Furthermore, the level of awareness and knowledge about cervical cancer, human papillomavirus (HPV) as its predominant cause, as well as screening services and treatment options in the general population in Ghana is low. Cervical cancer is mainly seen as a fatal condition [9–14].

Self-sampling, in conjunction with molecular HPV and/or oncoprotein testing, has so far been shown to be a reliable [15–17] and acceptable screening approach in hard-to-reach women in Sub-Saharan Africa as well as in high-income settings [18–21].

The uptake and scale-up of such emerging screening approaches largely depend on knowledgeable, motivated and capable health-care providers that are well respected by the communities they serve. A recent study from Tanzania showed that even though self-sampling was perceived as acceptable there was a high demand for nurse presence during self-sampling [22], which points to the importance of health-care providers for and during the self-sampling screening process.

In Ghana, health-care providers in hospitals and primary health facilities, including community health nurses (CHNs), constitute the most visible, front-line personnel providing health education to patients and the general population. Since CHNs play an integral role in educating women in the prevention of diseases, e.g. in antenatal services and child welfare clinics, they can influence cervical cancer screening adherence and health promotion among women [23]. Especially in rural areas, CHNs that live within communities are well respected and serve as important sources of health-related information. Thus, in the context of scaling up self-sampling services within a comprehensive cervical cancer strategy, health-care providers’ attitudes towards cervical cancer screening in general and self-sampling, in particular, are of key interest. We hypothesize that one strategy to enhance health-care workers’ acceptance and recommendation might be providing an opportunity for self-experience. The aim of the current study was to specifically explore female health-care providers’ personal perceptions of self-sampling for cervical cancer screening and implications their perceptions may have on promoting self-sampling within the general screening population.

### Study context

This study is part of the ACCESSING project (*A*dequate *C*ervical Cancer *C*apacity building, *E*ducation and *S*creening by New *S*cientific *IN*struments in *G*hana), a cross-sectional study investigating HPV prevalence via self-sampling and HPV testing in rural communities of the North Tongu district in Ghana [24]. The study also aimed at evaluating the feasibility of integrating cervical cancer screening via self-sampling into health-care services at the community level. Self-sampling acceptability results among targeted women of the general population in the ACCESSING trial are described elsewhere (manuscript in preparation).

In order to acquaint female health-care workers with the sampling method, a workplace screening program (occupational testing program) was set up. Female health-care providers were invited to self-collect a cervicovaginal sample with a self-sampling device and were interviewed to explore their perceptions of self-sampling as well as their potential role as screening advocates.

## Methods

A workplace screening program was conducted at a district hospital to acquaint female health-care providers with the sample collection method and thus enable them to guide women participating in the study on the self-sampling procedure. For this program, two different self-sampling devices were used; at first, the Delphi Screener (Rovers Medical Devices, The Netherlands) and later, due to non-availability of the Delphi Screener, the Evalyn® Brush (Rovers Medical Devices, The Netherlands). The Evalyn brush was also the device used for the community-based cross-sectional study.

### Study population and recruitment

A predefined number of 100 self-sampling kits were available for health-care providers as part of the workplace program for women working in the hospital as well as CHNs. There were no further eligibility criteria that had to be met apart from being a female health-care worker within the district. For the present study, we focussed our analysis on the female health professionals (n = 52) that had direct contact with patients, i.e. doctors, physician assistants, midwives, nurses, CHNs, ward assistants.

Health-care providers were made aware of the workplace program at clinical meetings, public events, as well as study education and training events. Self-sampling devices were given out by study nurses in the department of gynaecology at the district hospital upon request of interested health-care providers on a ‘first come, first served’ basis. Study nurses explained sampling technique to the participating female health-care providers. Together with the self-sampling device, a flyer was handed out that contained pictograms of the sampling procedure. We used the original manufacturer’s package insert [25].

All health-care providers who were HPV-positive were offered triage by cytology or Oncoprotein E6 test. All cytology positives were invited for clinical follow up at the district capital. Women received colposcopy, biopsy and treatment if indicated. Follow-up diagnostic and therapeutic measures were financed by study grants (grant by German Rotary Voluntary Doctors). Ethical clearance was obtained for sampling and interviews from the Ethical Review Committees of the Ghana Health Service (GHS-ERC: 05/05/13). Written informed consent was received from all participants.

### Study design

We chose a mixed-methods approach to assess acceptability and explore perceptions of self-sampling among workplace screening program participants. Mixed-methods approaches have become increasingly popular in recent years in the field of health sciences [26]. The approach is said to combine strengths of both quantitative and qualitative research paradigms [27]. Our design consisted of a quantitative survey, supplemented with one-to-one in-depth interviews to obtain insights into individual experiences and the effects of these experiences on health-care workers’ practice in terms of screening advocacy.

### Survey

Female staff members that had self-collected a sample between March 2014 and September 2015 as part of the workplace screening program filled in a simple survey after collecting the sample. The questionnaire was designed by the research team to assess the experiences of the health workers undergoing self-sampling. It was a short version of the questionnaire used in the ACCESSING study that was based on a questionnaire from a German self-sampling study by Deleré et al. [28] and adapted by the Ghanaian-German research team.

The questionnaire covered areas such as how easy and comfortable sampling were, how frequently they were likely to get screened in the future if the method was available, and their preference for self-sampling compared to speculum examination (see Appendix A for complete list of questions). Data entry was done on-site. Responses of the health workers with direct patient contact were analysed using SAS®, Version 9.2 (SAS Institute, North Carolina, USA). Descriptive statistical analyses were done using averages and ranges for continuous variables and frequencies and percentages for categorical variables. A Z-test was used to compare the proportions of women by stated intention to use self-sampling in the future, among those women who had previously had a speculum examination versus those that had not. The sample size was not specifically powered to detect differences between these groups.

### Interviews

For the qualitative part, 10 staff members with direct patient contact who had taken a self-sample were interviewed in September and October 2015. The semi-structured interview guide was designed by the Ghanaian-German research team and based on a literature review. It contained open-ended questions on experiences, perceptions, and implications of participating in the workplace program (see Appendix B). As the topic of self-sampling was considered to a sensitive topic, the Ghanaian-German research team decided on one-to-one interviews as an appropriate format to encourage participants to share their experience in a familiar and pleasant environment. Participants were assured that responses would be treated as confidential.

Before beginning interviews, we estimated that data saturation would be reached with 10 interviews. In order to include as many perspectives as possible on this potentially controversial topic, we chose a purposeful sampling strategy. Sampling was based on the principle of maximum variation sampling: female health-care providers having different professional profiles, training, and age were invited by one of the local study nurses face-to-face or over the phone prior to the interview. The interviews were conducted by the first author (female, German, MD), at the time of the study a senior medical student with prior training in qualitative research. Some of the interview participants were familiar with the interviewer prior to the interviews through clinical meetings and may have associated her with the ACCESSING research team. Interviews were held in quiet consulting rooms within the hospital premises with only the interviewer and the interview participant present. Interviews were conducted in English and audio-recorded. After each session, the interviewer filled in an interview protocol with information about atmosphere, interaction, main discussion points and needs for future questions [27]. The interviews were transcribed verbatim by the first author. The analysis was based on qualitative content analysis guidelines developed by Philipp Mayring, whereby the material is summarized and structured in a stepwise approach [29]. After reading the transcripts several times, analysis focussed on the passages where providers’ personal perceptions of self-sampling for cervical cancer screening and implications of their self-experience were discussed. Categories were formed in a deductive-inductive manner: research foci were based on the interview guide that served as a structure for the analysis. Categories and sub-categories emerged from a thematic analysis of the data. At first, relevant passages were paraphrased (micro coding), common themes were identified, and then themes were summarized under a heading (sub-category). When necessary, several levels of abstraction remained. The coding scheme was then applied to all interviews. It was developed by the first author and refined by the research team. While developing and applying the code system, memos were used to write down thoughts and associations for interpretation [30]. MAXQDA® 12 (VERBI Software GmbH, Berlin, Germany) software was used to support the organization and analysis of the interview material. To improve quality and validity of the analysis and to ensure intersubjectivity, coding and results were discussed within the research team in regular meetings and with an interdisciplinary group of qualitative researchers at Charité – Universitätsmedizin Berlin.

Interview participants were not paid but provided with a drink, snack, and a small gift for household use to compensate them for their time. After analysis of interviews, we compared the quantitative survey results that had been completed by health-care providers with interview results to validate findings, identify reasons, and implications.

## Results

The self-sampling cervical cancer workplace program was highly acceptable for female health-care providers. All 100 self-sampling kits were quickly handed out and additional health-care providers expressed interest in participation. Without specific targeting, health-care providers from all disciplines, i.e. doctors, physician assistants, nurses, midwives, and CHNs took part.

### Survey

All 52 female health-care providers with direct patient contact who took part in the workplace screening program filled in the questionnaire after self-sampling. Sociodemographic data are shown in Table 1.

**Table 1.** Sociodemographic data of survey participants

Variable	n = 52	Range
**Age** in years (mean)	36	23–59
**Profession** Ward assistant Community Health Nurse Nurse Midwife Physician Assistant Doctor	7 21 13 6 3 2	

96% (50/52) of health-care providers took the sample themselves and 2% (1/52) had the sample taken by a fellow health worker at the clinic (one study participant did not respond to this question and could not be followed-up to provide her answer). Of the 50 who took the sample themselves, 100% found it ‘Easy’ (9) or ‘Very Easy’ (41). In addition, 92% (46/50) felt ‘Very Comfortable’ and 8% (4/50) ‘Comfortable’. 83% (43/52) of the health workers indicated that they would get checked more often if the self-sampler has accuracy equivalent to screening by a clinician at a clinic. 98% (51/52) indicated they would prefer self-sampling if the accuracy of self-sampling are comparable to that determined by clinician cytobrush sampling.

With regard to those staff members who had previously had a pelvic examination (n = 22), 21 (95%) of them were comfortable or very comfortable with the self-sampler, and 100% indicated they would go for screening at the same frequency (23%) or more often (77%) in future if self-sampling is as accurate as sampling by a clinician. Among those who had never had a pelvic exam, 97% (29 out of 30) would go for screening at the same frequency or more often in the future if self-sampling is as accurate as sampling by a clinician. The difference between the percentages (100% versus 97%) of these two groups was not statistically significant (p-value for Z-test: 0.99).

### Interviews

The study population for the one-to-one interviews consisted of 10 women. Sociodemographic data are shown in Table 2. The mean duration of the interviews was 23 minutes, ranging from 16 to 28 minutes. One woman declined the interview invitation without giving a reason; another interviewee was recruited to replace her. Seven women indicated that they had previously undergone screening.

**Table 2.** Sociodemographic data of interview participants

Variable	n = 10		Range
**Age** in years (mean)	41		28–59
**Number of children**	1.3		0–3
**Profession** Ward assistant Community Health Nurse Nurse Midwife Nurse and Midwife Physician Assistant Doctor	1 3 2 1 1 1 1		

Table 3 summarizes research foci, categories and sub-categories that emerged.

**Table 3.** Research foci, emerging categories and sub-categories

Research focus	Categories	Sub-categories
Motivation to participate	Taking the opportunity as a woman	Meeting the fear of cervical cancer Appreciating the non-cost involved
	Seeing the benefits as a health care provider	Wanting self-experience to authentically counsel patients Improving instructional skills
Experiences of the self-sampling process	Preferring self-sampling over clinician sampling	Feeling/anticipating less pain Appreciating not being exposed to others Valuing privacy
Workplace and community implications	Gaining knowledge/awareness regarding HPV and cervical cancer	Feeling more knowledgeable about HPV, its transmission and cervical cancer Improving aseptic techniques Improving referrals
	Becoming advocates for cervical cancer screening	Encouraging patients to go for screening Explaining to community members Sharing of experiences

### Motivation to participate

Interview participants described various motives for participating in the self-sampling workplace program. The motives can be categorized as benefits for the individual woman and benefits for the work as health care provider.

#### Taking the opportunity as a woman

From a personal perspective, fear of cervical cancer emerged as a strong motivation. This was, in part, influenced by working in the gynaecology department, as one nurse explained:

“People come with problem of bleeding and their cervix like some protruding if – with the – if you like – if the speculum to check the – the thing – the cervix, the way it looks it scares me so that is the reason why I – I took part in the screening.” (Interview 5)

Furthermore, the fact that self-sampling was free of cost was mentioned by several respondents throughout the interviews and might have been an enabling factor for participation. One interview participant, who was about to retire, wanted to take the opportunity to know her HPV status before leaving the workplace.

#### Seeing the benefits as a health care provider

From a professional perspective, respondents stressed the importance of self-experience to enable effective counselling and instruction of patients with regard to self-sampling. One CHN described how she wanted self-experience to know the pain involved in the self-sampling procedure:

“Oh, I – I wanted to screen myself to have feelings about how the thing is, whether it will be painful or it won’t be painful, so that if I can tell someone that ‘Oh, it’s painful’ or not. So I did it myself.” (Interview 7)

A ward assistant indicated how self-experience was necessary to enable her to instruct patients on the self-sampling technique:

“When it came, we did it, we have to do it and know how it is before we explain it to other people. If you don’t do it and then you are explaining it, they will not understand. You doing it will let you tell them that it’s easier.” (Interview 2)

### Experiences of the self-sampling process

#### Preferring self-sampling over clinician sampling

Interview participants were positively disposed towards self-sampling as a new sampling technique that had not been widely used in the hospital before. There was a strong preference among respondents for self-sampling over clinician sampling. A minority indicated that they approved equally of both self-sampling and clinician sampling. The reasons for preferring self-sampling were less (anticipated) pain compared to a speculum examination and more privacy, the latter being particularly important in a hospital setting where the women may personally know the gynaecological staff, as one physician assistant explained:

“I think this one [self-sampling] is better – because the Pap smear, I have to come here and lie down for somebody to take the sample. Because there I think the privacy you are shy, you don’t want anybody to look at your private part or people you know around, your own colleagues, doing it for you. It’s better [if] you are in the comfort of your home and take your sample. So that one is better than the first one.” (Interview 3)

### Workplace and community implications

#### Gaining knowledge/awareness regarding HPV and cervical cancer

Some female health-care providers indicated that they gained more knowledge about the nature of HPV, its transmission (especially regarding the role of men), as well as treatment options. Some said that it was not more knowledge per se about HPV and cervical cancer but that their awareness regarding the disease, transmission, and treatment options increased. A midwife explained that after participating in the screening program she had a better understanding of HPV as a transmittable virus and paid more attention to aseptic techniques during delivery:

“Initially I was thinking you could have some signs and symptoms if you are harbouring that HPV. Yeah, but I have learnt that you may not have any […] signs and symptoms. And then I also learnt it could be transmitted through delivery when […] a delivery is being conducted on you. It could be transmitted not only through sexual intercourse but through delivery, too. And – yes, delivery. So that’s a new thing. Initially I was thinking it’s only through sexual intercourse. […] So that means our aseptic techniques should be perfect. So that we don’t transmit it in the hospital.” (Interview 8)

A community health nurse described how becoming familiar with the screening program would enable her to refer patients for cervical cancer screening:

“So through that [experience] I can also encourage somebody having a wart or sore around the vagina to go for such a screening so that, if maybe it might be – eh – cervical cancer, the person can have early treatment.” (Interview 7)

#### Becoming advocates for cervical cancer screening

There was consensus among the female health-care providers interviewed that they appreciated the self-sampling screening program and would recommend participation to their patients as well as family members and neighbours.

A physician assistant described her positive attitude towards the self-sampling screening program after her self-experience:

“It keeps you positive. If you know once you have participated in it and you know it’s a good thing – you – you, it makes you aware of everything and then you also teach your par – eh your patients, you counsel them about the good eh – how important the whole thing is. And you also encourage them to take part in it.” (Interview 3)

A midwife explained how she shared her experience with women living in her neighbourhood:

“I told the women that [self-sampling] is the simplest because you will […] not walk to the hospital, go and open your thighs for somebody to see what – whatever ever you have. But this one is very simple. They instruct you whatever – what you will do, they talk to you what you will do. You enter your room. You take your smear and bring [it] to them. So it’s – it’s so easy. We talk to them. We share [our] experience with them.” (Interview 1)

There was strong support among interviewees to explicitly recommend self-sampling for cervical cancer screening. One respondent said that her recommendation would depend on the woman’s situation – for example, whether she had a room of her own, i.e. enough privacy, to take the sample.

### Synthesis of results

The quantitative data showed that the vast majority of participants perceived self-sampling as easy, comfortable and preferable to Pap smear collection. The qualitative data supported these findings and elucidated the motives why female health-care providers were ready to participate in cervical cancer screening. Reasons for appreciating and preferring self-sampling were more privacy and more comfort associated with self-sampling. Furthermore, the fact that it was free of cost may have played an important role.

## Discussion

While there is a growing body of literature regarding uptake and barriers to uptake of cervical cancer screening [31–33], evidence on best practices in implementation of cervical cancer screening services, especially in rural Africa, is lacking [34]. Our study set out to explore perceptions and acceptability of self-sampling among female health-care providers, who are of key importance in screening programs.

In our study, we found that self-sampling for cervical cancer screening was highly acceptable to female health-care providers who took their personal sample themselves. The majority of female health-care workers indicated that they perceived self-sampling as easy and comfortable and preferred self-sampling over clinician sampling as a first-line cervical cancer screening test. Although less than half of participants had had a Pap smear before, most of the female health-care providers were familiar with Pap smear collection through their clinical work as doctors, nurses or midwives, since CHNs were trained on Pap smear collection as part of the main ACCESSING study. As a minority of health workers had previously had a Pap smear themselves, for most health-care workers, their judgement relied on their experiences as sample-takers.

Our interviews indicated that experiencing self-sampling may have several positive implications on their work as health-care providers: through self-experience, health-care providers felt that they had gained more knowledge and confidence to advise patients as well as family members and neighbours to get screened for cervical cancer. Setting up a workplace program that offers staff members the chance to get screened for cervical cancer on a regular basis may thus enhance the uptake and sense of ownership of a screening program by health-care providers, more so than if screening has to be paid for out-of-pocket. Health-care providers who may feel that their role is limited to being service providers – especially in study settings with partners from the Global North – may feel more appreciated and included. In the context of working towards scaling up self-sampling services, establishing personal acceptability among health-care providers could eventually translate to greater acceptability within the target population generally, through health-care providers’ (positive) testimonials. The evaluation of knock-on effects of health-care workers’ personal sampling experiences on the acceptance of self-sampling by women in the wider community was beyond the scope of the program and needs further research.

Our results also point to an improvement of quality and consistency of counselling as well as service provision, i.e. aseptic techniques, through increased awareness and knowledge about the nature of HPV and its transmission. However, the study was not designed to evaluate any changes in service provision, quality or consistency of counselling. This warrants further investigation in future studies.

The female health-care providers in our study reportedly acted as cervical cancer screening advocates. Through the workplace screening program, they had the opportunity of self-experience that increased their confidence and willingness to share their own experience in order to convince other women to get screened. This is particularly interesting as yet to be published results from focus group discussions with screening participants in five communities in the same district indicate that health-care providers – female providers in particular – are well respected and trusted when it comes to health promotion and education. These findings are in line with a qualitative study on Malaysian women reporting that many respondents said that they would agree to be screened (by Pap smear) if this was recommended by their health-care provider [35]. Moreover, a study of African American women found that health-care providers were influential through providing information on the importance of routine screening [36]. A qualitative study in Mozambique found that health educators should emphasize the benefits of screening (less pain, potential protection against future cancer, and lower rates of mortality) rather than focusing on the sexual cause of cervical cancer [37]. Health-care providers hence play a crucial role in advocating for cervical cancer screening and positive self-experience could increase their willingness and understanding of cervical cancer screening.

Our study is, to our knowledge, the first assessment of a formal cervical cancer workplace screening program. Notably, less than half of the study participants (22/52) had previously been screened for cervical cancer via pelvic examination. Studies from other countries also showed a low rate of cervical cancer screening among health-care providers [38–41]. Thus, besides the benefits in terms of intervention uptake in the population more broadly, setting up a workplace program could play an important part in providing health-care services to staff members.

Previous studies in Sub-Saharan African countries have shown that self-sampling for cervical cancer screening is widely acceptable to women [42,43]. One study from Cameroon found that some health-care providers are sceptical about self-sampling [44]. In our sample, however, the vast majority preferred self-sampling. This might be due to greater awareness and education about HPV and cervical cancer (screening) at this specific hospital and is possibly not generalisable to other regions of Ghana.

### Methodological considerations

The quantitative survey results demonstrated a high level of acceptability and positive attitudes towards the self-sampling program. The qualitative data supported these findings and yielded additional insights into reasons and implications. The combination of quantitative and qualitative methods has been gaining recognition and importance. In our case, by triangulating quantitative and qualitative data, results converged and thereby validated as well as complemented each other [45,46]. However, there may have been a selection bias in both methodological approaches, limiting the validity of the findings: the quantitative survey was completed by screening participants who had already accepted the screening invitation. Moreover, we were unable to interview non-participants as this was not covered by our ethical clearance. This selection bias may have led to an over-representation of positive perceptions of self-sampling and the workplace program in general, and an under-representation of negative perspectives. Negative perceptions and barriers to screening should be investigated in future studies, preferably at several points in time (pre- and post-intervention). Furthermore, the interviewer, a medical student from Germany, could have been seen as part of the ‘donors’ funding the screening project, and participants may therefore have presented a very positive perception of the program. Lastly, perceptions of targeted health-care providers are not necessarily representative of the perceptions of the general population. Results from the exploratory part of the qualitative component, touching upon reasons for participation and the implications of self-experience on clinical practice, were not covered by the quantitative part of the methodology. These elements could be added to future quantitative surveys to assess these phenomena on a larger scale.

To expand the benefit of screening to reach more women than the 2000 that took part in the ACCESSING study (of which this study is component), the local study team has established a local independent screening program as well as a cervical cancer prevention and training centre (see http://www.battorcervicalcentre.org/), which is promoted beyond the catchment area, to the whole of Ghana and the West African region.

From our study, we deduce a need for further research concerning the self-perception of (female) health-care providers as cervical cancer screening advocates. The mid- and long-term effects of such programs on the target population have to be critically assessed, also with regard to the cost-effectiveness of setting up a formal workplace program. Given the potential of HPV vaccination for primary prevention of cervical cancer, health-care providers’ attitudes towards it should also be evaluated. With the potential of self-sampling and/or self-testing for other curable sexually transmitted infections [47], there may be further opportunities for self-experience among health-care providers, e.g., within workplace programs.

## Conclusions

Self-experience of self-sampling for cervical cancer screening through workplace programs could constitute an important part of a cervical cancer screening strategy. This may be especially relevant for complex cervical cancer screening interventions that rely on health-care providers’ support in order to screen hard-to-reach women, and should thus be integrated in the planning, roll-out and extension of such interventions. Female health-care professionals can play a crucial role as cervical cancer screening advocates and this advocacy may be enhanced by experiencing screening themselves. Further research, for example, on potential effects of self-experience on the quality and consistency of counselling and screening, is necessary to evaluate the long-term effects and cost-effectiveness of such programs.

## Data Availability

The datasets generated and/or analysed during the current study are not publicly available due to privacy provisions but are available from the corresponding author on reasonable request. Datasets are available upon request from andreas.kaufmann@charite.de.

## Paper context

New approaches are needed to detect and cure cervical cancer, a major health threat to women especially in developing countries. Health-care providers are at the front line of screening services. We showed that self-experience of self-sampling within a formal workplace program can positively influence health-care providers’ perceptions of self-sampling. Funders and policymakers should thus consider incorporating self-experience for health-care providers into cervical cancer screening interventions.

## Appendix A. Acceptability questionnaire

**Table:**

1. Where sample was taken: **1**-Clinic **2**-CHPS Compound **3**-Home
2. Who took the sample **1**-Self-Unsupervised **2**-Self-Supervised **3**-Health worker
3. If you took the sample by yourself, please indicate how easy or difficult it was to use the self-sampler for self-sampling? **1**-Very Easy **2**-Easy **3-**Difficult **4-**Very difficult
4. If you took the sample by yourself, please indicate how comfortable you felt collecting your own sample with the self-sampler? **1**-Very ComforTable **2**-Somewhat comforTable **3-**Somewhat uncomfortable **4**-Very uncomfortable **5**-not applicable
5. If the sample was taken by a health worker, how comfortable was it? **1**-Very ComforTable **2**-Somewhat comforTable **3**-Somewhat uncomfortable **4-**Very uncomfortable **5**-Not applicable
6. Prior to this screening, had a health professional ever taken your sample during a pelvic examination? **1**-Yes **2**-No
7. *If you answered ‘Yes*’, how comfortable did you feel when the health professional collected your samples at your last pelvic exam? **1**-Very ComforTable **2**-Somewhat comforTable **3**-Somewhat uncomfortable **4-**Very uncomfortable **5**-Don’t remember
8. If the self-sampler works as well as going to the doctor, would you get checked more often, less often or about the same? **1**-More often **2**-The same **3**-Less often.
9. If both sampling by speculum with brush and by sampling with self-sampler can determine your risk of cervical cancer equally, which one would you prefer? **1-**Sampling with speculum and brush **2**-Sampling with the self-sampler

## Appendix B. Semi-structured interview guide

- How did you hear about the self-sampling workplace program?
- Could you please describe your decision to participate in the screening program?
- Could you please describe your experience taking a sample with the self-sampling device?
- What is your overall opinion on self-sampling for cervical cancer screening?
- Which screening technique would you prefer? Why?
- How might participation in this self-sampling program influence your work with patients?
- Would you recommend self-sampling to other women?
